# Anatomical plantar pressure masking and foot models: potential for integration with marker position systems

**DOI:** 10.1186/1757-1146-5-S1-O29

**Published:** 2012-04-10

**Authors:** Claudia Giacomozzi, Julie Stebbins, Alberto Leardini

**Affiliations:** 1 Dept. of Technology and Health, Italian National Institute of Health (ISS), Rome, Italy; 2 Nuffield Orthopaedic Centre, Oxford, UK; 3 Istituto Ortopedico Rizzoli, Bologna, Italy

## Background

Investigation of local foot loading using baropodometry is highly relevant in both research and clinical settings. In order to reliably associate local pressure data with foot function and structure, anatomy-based masking of footprints is recommended, especially when foot anatomy or footprints are significantly altered. Previous studies combining baropodometry with stereophotogrammetry have shown the value of this methodology in specific, prototype-based situations [1,2]. This study thoroughly investigates the potential of this method.

## Materials and methods

A set of regular footprints from young healthy volunteers was acquired under controlled conditions by using commercial 3D kinematic tracking systems and pressure mats. The Oxford kinematic foot model [3] was used for medio-lateral regionalisation of the foot – clinically relevant for clubfoot and flatfoot –, the Rizzoli model [4] for longitudinal regionalisation, to clearly distinguish metatarsal from toe or midfoot loading.

## Results

100 footprints from 20 volunteers have been processed so far for the Oxford model (processing still ongoing for the Rizzoli model). For medial and lateral hindfoot, and for medial and lateral forefoot, differences from a proper geometry-based masking were 3.4-3.9% (vertical force), 0.7-2.7% (peak pressure), 1.6-4.5% (mean pressure), 2.1-3.8% (area); midfoot differences rose to 5.2% and 9.7% for peak and mean pressure. However, none of the differences were statistically significant.

## Conclusions

With a correct marker positioning, and an appropriate calibrated pressure mat (accuracy error <5%, spatial resolution 4sens/cm^2^), the method was validated with respect to a proper geometrical selection (differences <5%). The effect and clinical relevance of lower spatial resolution, marker positioning errors and use of clusters instead of skin markers, is also being investigated.

**Figure 1:**
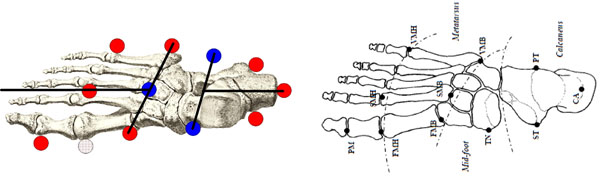
Projections of marker set onto the footprint obtained by applying the Oxford model (left) and the Rizzoli model (right).
